# The Differential Effects of Anesthetics on Bacterial Behaviors

**DOI:** 10.1371/journal.pone.0170089

**Published:** 2017-01-18

**Authors:** Matthew Chamberlain, Sophia Koutsogiannaki, Matthew Schaefers, Hasan Babazada, Renyu Liu, Koichi Yuki

**Affiliations:** 1 Department of Anesthesiology, Perioperative and Pain Medicine, Boston Children’s Hospital, Boston, Massachusetts, United States of America; 2 Department of Anesthesiology and Critical Care, University of Pennsylvania, Philadelphia, Pennsylvania, United States of America; The University of Tokyo, JAPAN

## Abstract

Volatile anesthetics have been in clinical use for a long period of time and are considered to be promiscuous by presumably interacting with several ion channels in the central nervous system to produce anesthesia. Because ion channels and their existing evolutionary analogues, ion transporters, are very important in various organisms, it is possible that volatile anesthetics may affect some bacteria. In this study, we hypothesized that volatile anesthetics could affect bacterial behaviors. We evaluated the impact of anesthetics on bacterial growth, motility (swimming and gliding) and biofilm formation of four common bacterial pathogens *in vitro*. We found that commonly used volatile anesthetics isoflurane and sevoflurane affected bacterial motility and biofilm formation without any effect on growth of the common bacterial pathogens studied here. Using available *Escherichia coli* gene deletion mutants of ion transporters and *in silico* molecular docking, we suggested that these altered behaviors might be at least partly via the interaction of volatile anesthetics with ion transporters.

## Introduction

Anesthesia is a critical component of surgical procedures, and volatile anesthetics have been the mainstay of drugs for more than a century. Although the anesthetic mechanism of volatile anesthetics has not been completely delineated yet, the general consensus is that they are promiscuous and work by directly interacting with several ion channels in the central nervous system [1]. Intravenous anesthetics also work via ion channels but have more specific targets, such as the anesthetic effect of propofol via gamma-aminobutyric acid type A (GABA_A_) receptors [2]. The primary goal of anesthesia providers is to make patients comfortable during surgery, and the effect of anesthetics on different organisms such as bacteria is not usually taken into consideration in the clinical setting. However, it is not rare to anesthetize patients with active bacterial infections. Changes in bacterial behavior after exposure to anesthetics at clinically relevant concentrations, if any, could have significant, clinical implications.

The interest in the effect of anesthetics on bacteria is not new, but studies are rather limited. Previous research solely studied the effect of anesthetics on bacterial growth, as the association between the severity of bacterial infection and higher bacterial load is intuitively clear, and has been supported by the literature [3–5]. Most of the previous studies suggest that volatile anesthetics at clinically relevant concentrations do not affect bacterial growth as summarized in **Table 1**[6–10]. In contrast, the effect of anesthetics on other bacterial behaviors including motility and biofilm formation has seldom been studied. Many bacteria are generally motile and some bacteria swim in liquid or swarm over solid surfaces using flagella [11]. While swarming is a form of bacterial movement in a group, swimming is an individual bacterial movement [12]. In addition, some bacteria can glide over solid surfaces without flagella. Bacteria can also accumulate and form non-motile multicellular aggregates. These aggregates of bacteria connected by extracellular polysaccharides are called biofilm. Biofilm can be more resistant to antibiotics than planktonic bacteria cells and contributes to persistent infection [13].

**Table 1:** The previous studies of effects of anesthetics on bacterial growth.

Drug tested	Bacteria	Duration of exposure	Results	References
3% Sevoflurane, 60% Nitrous oxide	Pseudomonas aeruginosa Acinetobacter lwoffii, Staphylococcus aureus	3 hrs	No change in S.aureus, very small increase in A.lwoffii and P.aeruginosa under 3% sevoflurane, more than 2 fold increase in A.lwoffii and P. aeruginosa under 60% nitrous oxide	[10]
Isoflurane (2%)	Escherichia coli, Staphylococcus aurerus	2 hrs	No chance	[9]
Isoflurane (21–25%)	Staphylococcus aureus, Streptococcus pneumoniae, coliform bacteria	16 hrs	No effect	[8]
halothane (2–5%)	Staphylococcus aureus, Escherichia coli	4 hrs	No effect	[7]
Isoflurane (1.1–2.3%), halothane (0.8–1.5%)	Psedumonas aeruginosa	4 hrs	Reduction in growth under both isoflurane and halothane	[14]
Halothane (1–10%)	Escherichia coli, Bacillus licheniformis, Staphylococcus albus, Micrococcus lysodeikticus	24 hrs	Effect seen only at > 5% of halothane	[6]

Here we examined the effect of three commonly used anesthetics isoflurane, sevoflurane and propofol on four bacteria species; Gram-positive *Staphylococcus aureus* (*S*. *aureus*) and *Enterococcus faecalis* (*E*. *faecalis*) and Gram-negative *Escherichia coli* (*E*.*coli*) and *Pseudomonas aeruginosa (P*. *aeruginosa)*. *S*. *aureus* and *E*. *faecalis* are non-flagellated bacteria and can form biofilm [15, 16]. *S*. *aureus* is known to glide [17], while *E*. *faecalis* is non-motile [18]. *E*. *coli* and *P*. *aeruginosa* are flagellated bacteria with swimming motility and also form biofilm [19–21]. First, we tested bacterial growth. Then, we tested the effect of anesthetics on swimming motility capability of *E*. *coli* and *P*. *aeruginosa*, on gliding motility *of S*. *aureus* and on biofilm formation in all four bacteria.

## Materials and Methods

### Bacterial species and plasmids

We used *E*. *coli* (K12 strain), *S*. *aurerus* (Newman strain), *E*. *faecalis* (strain 12030), and *P*. *aeruginosa* (strain PA14). *E*.*coli* K12 strains were purchased from E. coli Genetic Stock Center (CGSC; New Haven, CT, USA)[22]. *E*. *faecalis* and *P*. *aeruginosa* strains were kindly provided by Dr. Gregory Priebe (Boston Children’s Hospital, Boston, MA, USA). *S*. *aureus* Newman strain was kindly provided by Dr. Timothy Foster (Trinity College Dublin, Dublin, Ireland). In addition, plasmids used here (pKD3, pKD13, pKD46 and pCP20) [22] were purchased from *E*. *coli* Genetic Stock Center. All the bacteria used here were shown in **Table 2**.

**Table 2:** Bacterial strains used in the study.

Bacteria strains or plasmids	Description of genotype	Reference
**Bacteria**		
*E*. *coli* K12 (MG1655)	*E*. *coli* K12 Parent strain, wild-type	[22]
*E*. *coli* K12 (JW5012)	*clcA* deletion mutant, Kan^R^	[22]
*E*. *coli* K12 (JW5263)	*clcB* deletion mutant, Kan^R^	[22]
*E*. *coli* K12 (JW0018)	*nhaA* deletion mutant, Kan^R^	[22]
*E*. *coli* K12 (JW1175)	*nhaB* deletion mutant, Kan^R^	[22]
*E*. *coli* K12 (JW1207)	*chaA* deletion mutant, Kan^R^	[22]
*E*. *coli* K12 (JW3313)	*kefB* deletion mutant, Kan^R^	[22]
*E*. *coli* K12 (JW0046)	kefC deletion mutant, Kan^R^	[22]
*E*. *coli* K12 (JW3251)	*trkA* deletion mutant, Kan^R^	[22]
*E*. *coli* K12 (JW0454)	mscK deletion mutant, Kan^R^	[22]
*E*. *coli* K12 (JW1908)	*fliC* deletion mutant, Kan^R^	[22]
*E*. *coli* K12 (Triple mutant)	*nhaA*, *nhaB*, *clcB* deletion mutant, Kan^R^, Cat^R^	This study
*E*. *coli* K12 (double mutant 1)	*nhaA*, *nhaB* deletion mutant, Kan^R^	This study
*E*. *coli* K12 (double mutant 2)	*nhaA*, *clcB* deletion mutant, Kan^R^, Cat^R^	This study
*S*. *aureus* Newman	CC8 clinical isolates	TF
*E*. *faecalis* 12030	Clinical isolates	GP
*P*. *aeruginosa* PA14	Multi host pathogen	GP
**Plasmid**		
*pCP20*	Fln recombinase gene (+)	[22]
*pKD3*	Cat resistance gene flanked by FRT	[23]
*pKD13*	Kan resistance gene flanked by FRT	[23]
*pKD46*	Lambda red recombinase expression plasmid	[23]

*GP = obtained from Dr. Gregory Priebe (Boston Children’s Hospital), TF = obtained from Dr. Timthy Foster (Ireland)

### Bacterial growth

Overnight cultures of all four bacterial species were diluted with tryptic soy broth (TBS) to the optical density 600 (OD_600_) of 0.05. Bacteria were grown at 37°C for 4 or 6 hours with or without anesthetics. Volatile anesthetics were exposed to bacteria using an airtight chamber at clinically relevant concentrations as indicated in each experiment. Propofol was dissolved in dimethyl sulfoxide (DMSO). The final concentration of DMSO in propofol sample and its control was 0.1%. The concentration of propofol used in this study (50 μM) is significantly above the clinically relevant concentration [24]. After bacterial culture for the indicated time, samples were serially diluted, and plated on tryptic soy agar (TSA) plates and incubated at 37°C overnight for determination of colony forming units (CFU)/mL.

### Swimming assay on agar plate

A 10-μL drop of overnight *E*. *coli* and *P*. *aeruginosa* cultures were added to plates consisting of lysogeny broth (LB) with 0.3% agar. *E*. *coli* was incubated for 12 hours and *P*. *aeruginosa* was incubated for 24 hours at 37°C with or without anesthetics. At the end of incubation, pictures of the swimming formation were taken and swimming area was measured using Image J (National Institutes of Health, Besthesda, MD, USA). Isoflurane and sevoflurane were exposed in an air-tight chamber. Control samples were also incubated in an airtight chamber to match the condition.

### Swimming examination under microscope

*E*. *coli* K12 cultures were diluted into 2% of poly-ethylene glycol (PEG) aqueous solution as previously described [25]. A group of *E*. *coli* cells were exposed to isoflurane for 5 minutes. Movement was recorded and tracked using MetaMorph software (Molecular Devices, Sunnyvale, CA, USA).

### Flagellin expression analysis using Western blot

We used *E*.*coli* K12 parent strains and mutants lysates to test flagellin expression. Some of *E*.*coli* parent strain cells were exposed to isoflurane for indicated durations. *E*. *coli* cells were suspended in 10mM Tris-HCl, pH 8.0, 100 mM NaCl, 1 mM MgCl_2_ as previously described [26]. After 10-second of sonication, the concentration of bacterial lysates was measured using BCA protein assay (Thermo Fisher Scientific, Waltham, MA, USA). Samples of the same weight were loaded on SDS-PAGE gel and blotted to nitrocellulose membrane. The membranes were probed with rabbit anti-flagellin antibody (abcam, Cambridge, UK) or goat anti-glyceraldehyde 3-phosphate dehydrogenase (GAPDH) antibody (Genscript, Piscataway, NJ, USA). Anti-rabbit IgG-horseradish peroxidase (HRP) conjugate (Cell Signaling, Danvers, MA, USA) or anti-goat IgG-HRP conjugate (Thermo Fischer Scientific) was used as the secondary antibody, respectively. Signal was detected with chemoluminescence (Thermo Scientific).

### *S*. *aureus* gliding assay

A 10-μL drop of overnight *S*. *aureus* cultures were added to 0.3% LB agar plates with or without anesthetics for 19 hours. At the end of incubation, pictures of the gliding formations were taken and their areas were measured using Image J.

### Biofilm assay

Following overnight growth of bacteria at 37^°^C, 100 μL of bacteria at OD_600_ of 0.05 was aliquoted to 96-well U-bottom plates and incubated at 37°C for 48 hours with or without anesthetics as previously described [27, 28]. Isoflurane and sevoflurane were exposed to samples as described in the swimming assay using an air-tight chamber. Control samples were also incubated in an air-tight chamber to match the condition. Wells were then washed and stained with 150 μL of 0.1% of crystal violet. Following incubation at room temperature for 10 minutes, plates were rinsed with water and dried for a few hours. Then, 30% of acetic acid was added to each well to solubilize the crystal violet, which was transferred to a new flat-bottomed plate. Absorbance was read at 590 nm using Versamax Tunable Microplate Reader (Molecular Devices, Sunnyvale, CA, USA).

### Search of *E*. *coli* ion transporters

EcoCyc (http://ecocyc.org) [29] was used to search for ion transporters in *E*.*coli* K12 MG1655 strain.

### Gene deletion

Gene deletion was performed as previously desribed [23]. *clcB* deletion mutant was transformed with pCP20 plasmid to remove kanamycin resistant cassette. Then, cells were transformed with pKD46. Red recombinase was induced with L-arabinose (Fischer scientific), and then competent cells were prepared and transformed with PCR products for *nhaB* gene deletion. PCR product was made using pCD13 as a template to have kanamycin resistant cassette. Similarly, PCR products made using pKD3 as a template to delete nhaA gene with chloramphenicol cassette were transformed to make *nhaA*(-)*nhaB*(-)*clcB*(-) mutant. Primer sets was refered to the paper by Baba et al [22].

### Docking of isoflurane onto *E*.*coli* protein NhaA

Using previously reported structures in Protein Data Bank (PDB) (http://www.rcsb.org) of NhaA protein (PDB ID: 1ZCD), we performed the docking of isoflurane. *Autodock* software (The Scripps Research Institute; La Jolla CA, USA) was used as a docking software to identify the top ranking docked site of isoflurane.

### Statistical analysis

All the statistical analyses were performed using Prism 6 software (GraphPad Software, Inc., La Jolla, CA, USA). Statistical analyses used were included in the corresponding figure legends. *P* < 0.05 was considered to be statistically significant.

## Results

### Isoflurane, sevoflurane and propofol did not affect bacterial growth

**Fig 1:**
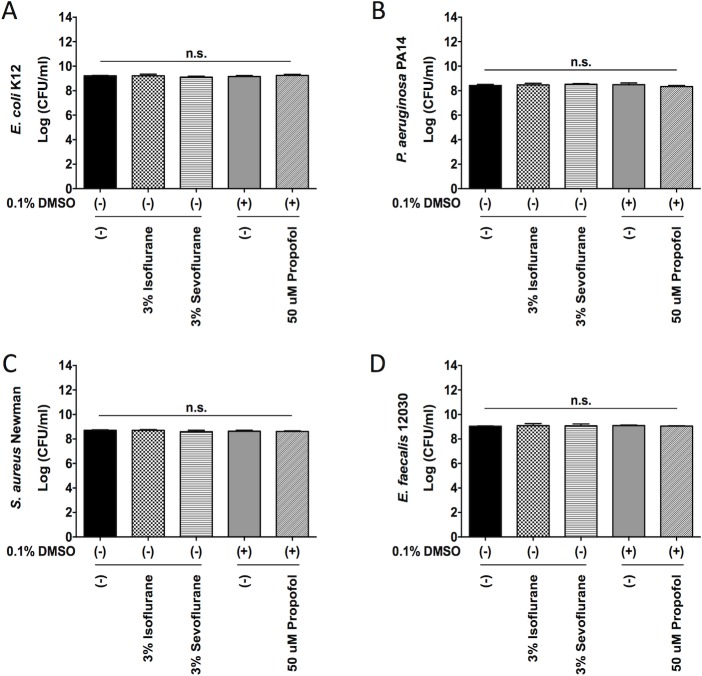
The effect of anesthetics on bacterial growth. The growth of *E*. *coli* (A), *P*. *aeruginosa (B)*, *S*. *aureus* (C), and *E*. *faecalis* (D) under isoflurane, sevoflurane or propofol at various concentrations is shown. Data are shown as mean +/- S.D. of 4 replicates. Statistical analysis was performed using one-way analysis of variance with Bonferroni *post hoc* analysis. No statistical significance was noted. n.s. = not significant. CFU = colony forming unit.

First, we tested the effect of isoflurane, sevoflurane at clinical relevant concentrations and propofol at a supraclinical concentration on the growth of four bacterial species. We used TSB as a growth media, which is considered as a medium rich in nutrition and protein content, similar to some human exudates [14]. We did not observe any significant difference in bacterial growth between the anesthetics-exposed group and the non-exposed group (**Fig 1**). This is in line with the majority of available literature demonstrating that volatile anesthetics at clinically relevant concentrations do not affect bacterial growth (**Table 1**).

### Isoflurane and sevoflurane diminished bacterial swimming

**Fig 2:**
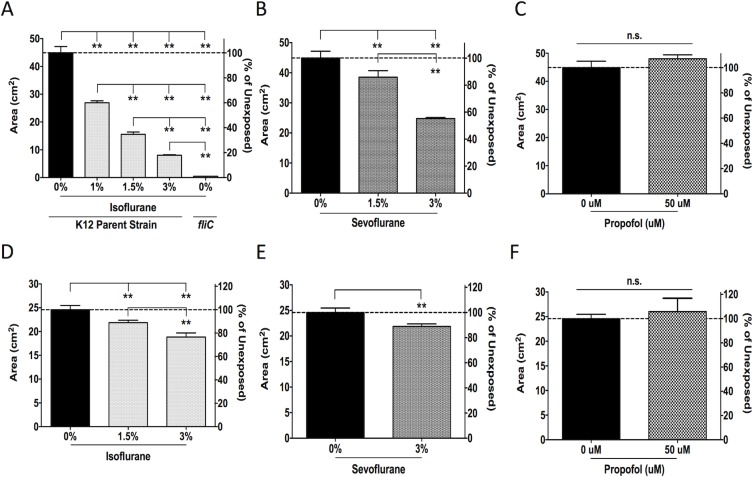
The effect of anesthetics on bacterial swimming. Areas (cm^2^) of motility halos formed by *E*. *coli* K12 *parent strain* (A-C) and *P*. *aeruginosa* (D-F) exposed to isoflurane, sevoflurane or propofol at various concentrations are shown. *E*. *coli* K12 *fliC* deletion mutant, which lacks flagellin, was included as a control strain with no motility in (A). Data shown as mean ± SD; n = 4 for all conditions. Statistical analysis was performed using one-way analysis of variance with Bonferroni *post hoc* analysis for A, B and D. C, E, and F were analyzed using an unpaired t-test. * and ** denote *p* < 0.05 and *p* < 0.01, respectively. n.s. = not significant.

We tested the effect of anesthetics on swimming of flagellated bacteria *E*.*coli* and *P*. *aeruginosa*. Both isoflurane and sevoflurane significantly reduced *E*.*coli* swimming at all the concentrations tested. The reduction in swimming was dose-dependent and treatment with 3% isoflurane and sevoflurane resulted in 80% and 50% reduction compared to untreated *E*. *coli*, respectively (**Figs 2A and 2B and S1**). The *fliC* deletion mutant served as a non-motile control strain (**Fig 2A**). Propofol, on the other hand, did not affect *E*. *coli* swimming (**Figs 2C and S1**). The swimming of *P*. *aeruginosa* was also reduced by isoflurane and sevoflurane, but not by propofol (**Figs 2D–2F and S1**). The effect of isoflurane and sevoflurane on *P*. *aeruginosa* swimming was more modest than that on *E*. *coli*.

### Isoflurane attenuated *E*.*coli* motility without the reduction of flagellin expression

**Fig 3:**
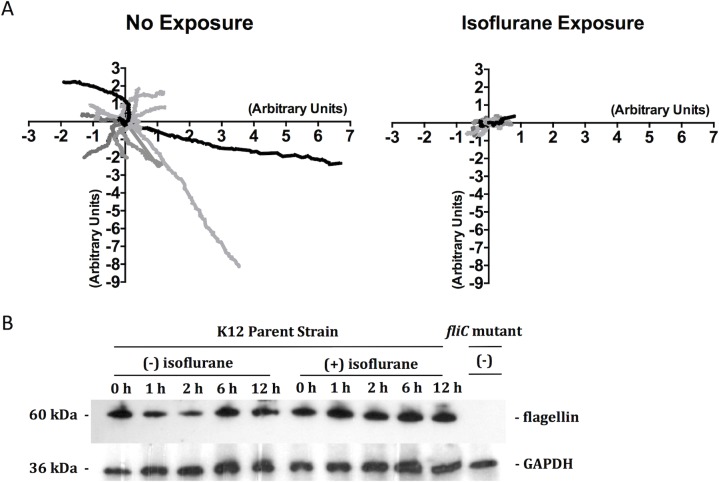
The effect of isoflurane on *E*. *coli* motility and flagellin expression. (A) The motility of *E*. *coli* K12 parent strain was observed in 2% PEG aqueous solution. A group of *E*. *coli* cells were exposed to isoflurane for 5 minutes before subjecting to microscope analysis. Movement over 10 second was tracked using MetaMorph software. (B) Flagellin expression was examined with or without isoflurane exposure for the indicated durations. GAPDH was used as a loading control [46]. *fliC* deletion mutant was used as a negative control of flagellin expression

We also evaluated the movement of *E*. *coli* under microscope. A brief exposure to isoflurane attenuated *E*. *coli* movement (**Fig 3A**), suggesting that the attenuation of *E*. *coli* motility by isoflurane occurred immediately and was unlikely to be caused by the change of protein expression such as flagellin expression. As expected, isoflurane exposure did not attenuate flagellin expression (**Fig 3B**).

### Isoflurane enhanced *S*. *aureus* motility

**Fig 4:**
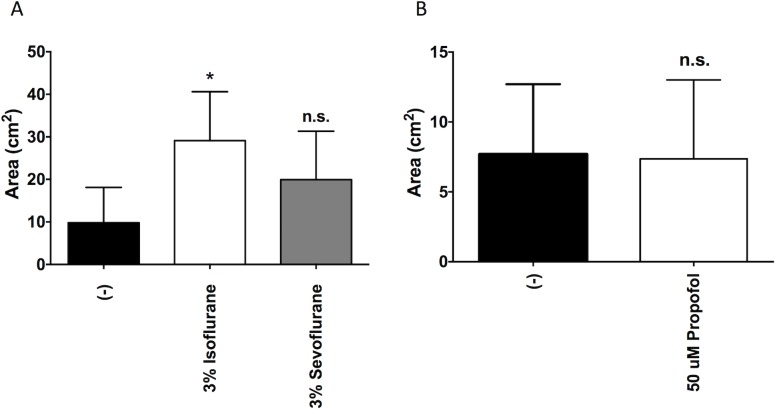
The effect of anesthetics on *S*. *aureus* gliding. Areas (cm^2^) of gliding formation of Newman *S*. *aureus* after exposure to isoflurane or sevoflurane (A) or propofol (B) for 19 hours are shown. Data shown as mean ± SD; n = 4 for A; n = 8 for B. Statistical analysis was performed using one-way analysis of variance with Bonferroni *post hoc* analysis for A. B was analyzed using an unpaired t-test. * denotes p<0.05. n.s. = not significant.

*S*. *aureus* is a non-flagellated bacteria but moves by gliding [17]. We found that isoflurane significantly increased motility of *S*. *aureus* approximately 3-fold, but sevoflurane and propofol did not (**Fig 4**).

### Isoflurane and sevoflurane exposure increased bacterial biofilm formation

**Fig 5:**
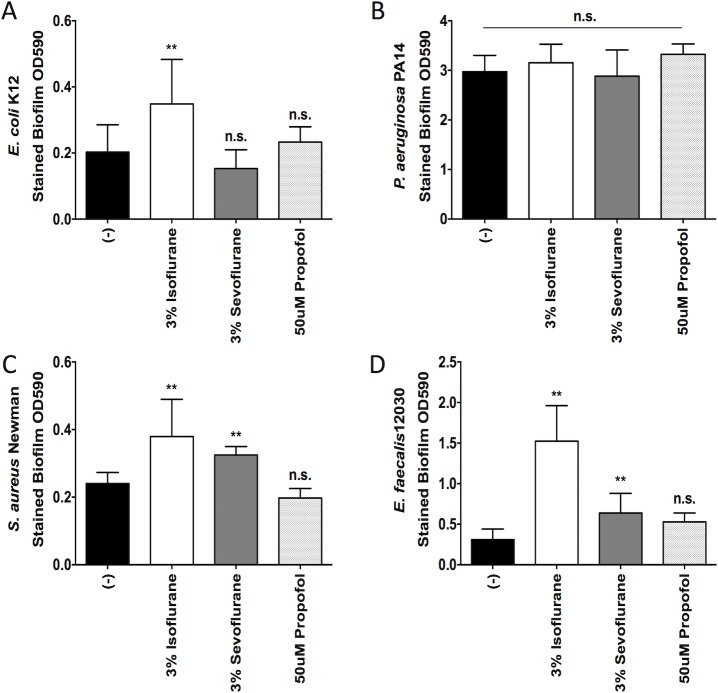
The effect of anesthetics on bacterial biofilm formation. The biofilm formation of *E*. *coli* (A), *P*. *aeruginosa* (B), *S*. *aureus* (C), and *E*. *faecalis* (D) after exposure to isoflurane, sevoflurane or propofol is shown. Data are shown as mean +/- S.D. of 8–12 replicates. Statistical analysis was performed using one-way analysis of variance with Bonferroni *post hoc* analysis. * and ** denote *p* < 0.05 and *p* < 0.01 of non-exposure vs exposure, respectively. n.s. = not significant.

We also evaluated the effect of anesthetics on biofilm formation by all four bacteria. *P*. *aeruginosa*, *S*. *aureus* and *E*. *faecalis* showed significantly more biofilm formation than *E*. *coli* (**Fig 5**). We found that isoflurane enhanced biofilm formation of *S*. *aureus* and *E*. *faecalis* by about 60% and 390% respectively, not *P*. *aeruginosa* (**Fig 5**). Isoflurane also enhanced biofilm formation by *E*. *coli* by 73%. In addition, sevoflurane enhanced biofilm formation by only *S*. *aureus* and *E*. *faecalis* by about 33% and 100% respectively. Propofol had no significant effect on biofilm formation.

### The potential interaction of isoflurane with *E*. *coli* ion transporters

**Fig 6:**
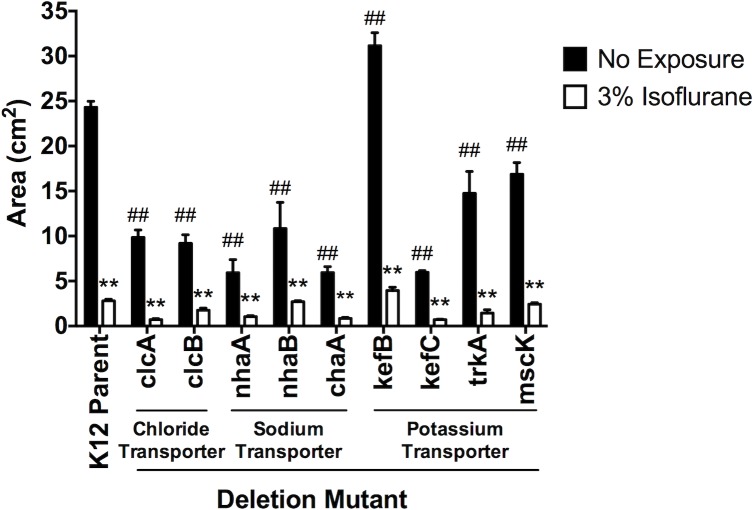
The effect of isoflurane on swimming of *E*. *coli* K12 parent strain and ion transporter deletion mutants. The effect of isoflurane on the swimming of *E*. *coli* K12 strains is shown. Data are shown as mean +/- S.D. of 4 replicates. Statistical analysis was performed using two-way analysis of variance with Bonferroni *post hoc* analysis for differences between non-exposure and exposure groups. * and ** denote p<0.05 and p<0.01, respectively. For differences between deletion mutants and the K12 parent strain within the non-exposure group, statistical analysis was performed using one-way analysis of variance with Bonferroni *post hoc* analysis. # and ## denote p<0.05 and p<0.01, respectively.

Mammalian ion channels and bacterial ion transporters are shown to be evolutionarily connected [30]. Because volatile anesthetics isoflurane and sevoflurane are considered to act via ion channels in the central nervous system, we hypothesized that they affected bacterial behaviors via their ion transporters. The genome of *E*. *coli* K12 strain has been completely sequenced [31], and a library of single gene deletion mutants has been created in this strain [22]. We identified nine ion transporters in *E*.*coli* K12 strain using EcoCyc database (**Table 3**) and we tested our hypothesis using nine ion transporter deletion mutants with or without isoflurane exposure. Because isoflurane reduced swimming of E. coli and we hypothesized that isoflurane would interact with ion transporters in *E*. *coli*, we predicted some of these ion transporter deletion mutants would show defect in swimming. As predicted, we found that the majority of ion transporter deletion mutants showed defect in swimming (**Fig 6**). In addition, these ion transporter mutants did not show any reduction in flagellin expression (**S2 Fig**). The majority of mutants had higher flagellin expression than that of parent strain. This result suggested that their swimming defect was not due to defect in flagellin expression. It is a general consensus that anesthetic effect by volatile anesthetics is through their interaction with multiple ion channels [32]. Therefore, we predicted that swimming impairment by isoflurane would derive from its interaction with multiple ion transporters. The addition of isoflurane to most of these mutants further attenuated swimming, supporting our prediction that multiple isoflurane target sites would exist in *E*. *coli* (**Fig 6**). When we evaluated the percentage of reduction in swimming under isoflurane, *clcB*, *nhaA* and *nhaB* deletion mutants showed less reduction by isoflurane than the parent strain (parent strain 88.5%, *clcB*(-) 80.8%, *nhaA*(-) 82.4%, and *nhaB*(-) 75.0%)(**S3 Fig**), suggesting that these three ion transporters could be isoflurane targets. We tested swimming of double and triple mutants. They swam significantly less than K12 parent strain (**S4A Fig**), suggesting that they were critical for swimming. When we compared the effect of isoflurane on *clcB*(-)*nhaA*(-)*nhaB*(-) mutant, swimming of this triple mutant was less reduced under isoflurane (parent strain 91.5%, triple deletion 6.4%), suggesting that they would be likely targets for isoflurane (**S4B Fig**). However, the reduction of the motility by isoflurane may be difficult to estimate because the triple mutant cells were already nearly non-motile without isoflurane.

**Table 3:** Known ion transporters in *E*.*coli* K12.

Classification	Gene name
Chloride ion transporter	*clcA*
	*clcB*
Sodium ion transporter	*nhaA*
	*nhaB*
	*chaA*
Potassium ion transporter	*MscK*
	*KefB*
	*KefC*
	*trkA*

### Isoflurane increased biofilm formation in *E*.*coli* K12

**Fig 7:**
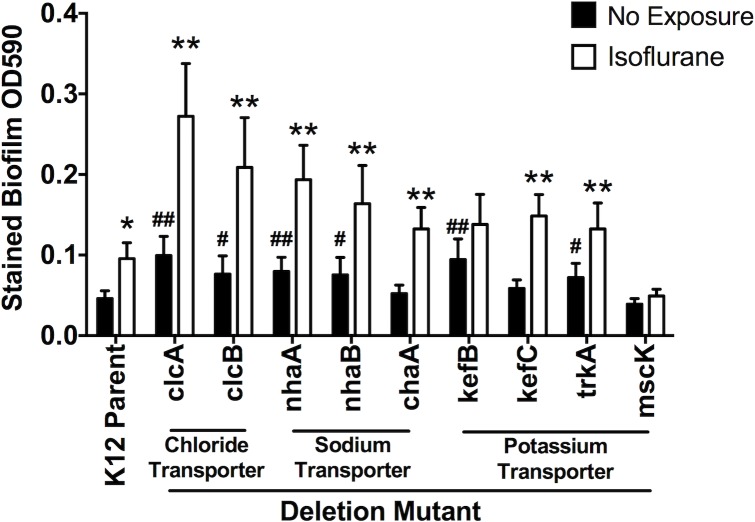
The effect of isoflurane on biofilm formation of *E*. *coli* K12 parent strain and ion transporter deletion mutants. The effect of isoflurane on biofilm formed by *E*. *coli* K12 strains is shown. Data are shown as mean +/- S.D. of 8 replicates. Statistical analysis was performed using two-way analysis of variance with Bonferroni *post hoc* analysis for differences between non-exposure and exposure groups. * and ** denote p<0.05 and p<0.01, respectively. For differences between deletion mutants and the K12 parent strain within the non-exposure group, statistical analysis was performed using one-way analysis of variance with Bonferroni *post hoc* analysis. # and ## denote p<0.05 and p<0.01, respectively.

Because isoflurane increased biofilm formation by *E*. *coli*, we predicted that some of ion transporter deletion mutants would show increased biofilm formation than its parent strain (**Fig 7**). We found that *clcA*, *clcB*, *nhaA*, *nhaB*, *kefB*, and *trkA* deletion mutants had higher biofilm formation than its parent strain, and isoflurane further enhanced biofilm formation by these mutants (**Fig 7**). In addition, none of these mutants did not have any defect in growth (**S5 Fig**). Isoflurane targets are likely to be ones that are involved in both swimming and biofilm formation, not growth, and *clcB*, *nhaA*, and *nhaB* fitted for this category.

### Isoflurane has predicted binding site in ion transporters in *E*. *coli*

**Fig 8:**
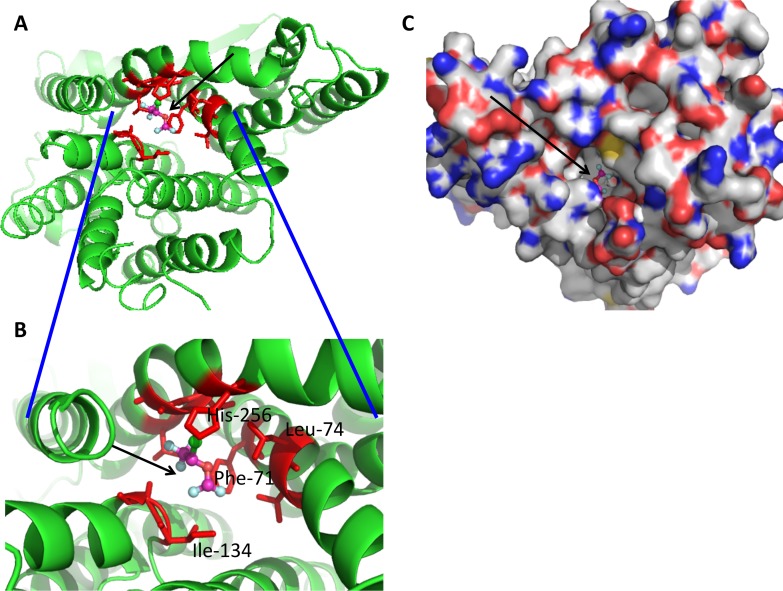
Docking simulation of isoflurane onto NhaA protein (PDB ID: 1ZCD). Docked image of isoflurane is shown (arrow). In isoflurane, light blue; floride, green; chloride and orange; oxygen. Red residues are within 4 angstroms of isoflurane. (A) Docked image in cartoon. (B) Blowout image of docked site. (C) Docked image in surface.

Using available structure of *E*.*coli* ion transporters, we sought for potential isoflurane binding site There were no reported structures of ClcB and NhaB. Protein structures for NhaA were reported [33–35]. Using NhaA structure, we performed docking simulation for the potential binding site (**Fig 8**). The trifluoromethyl head of isoflurane formed a hydrophobic interaction with Phe-71, Ala-135, Ala-260 and Leu-264, and the difluoromethyl head formed with Phe-71 and Ile-134. Amino acid residues within 4 angstrom from docked isoflurane were Phe-71, Asp- 133, Ile-134, Ala-135, Phe-136, Ala-137, Gly-139, His-256, Val-259, Ala-260 and Leu-264. The functional role of NhaA amino acid residues was reviewed in detail [36]. Asp-133 is at the iron binding site and His-256 is essential for pH signal transduction, suggesting that isoflurane docking site will be functionally critical for NhaA activity. This supports the idea that isoflurane affects ion transporter function.

## Discussion

Here we demonstrated that 1) volatile anesthetics tested at clinically relevant concentrations did not affect growth of four common bacterial pathogens, 2) isoflurane and sevoflurane significantly reduced swimming of *E*. *coli* and *P*. *aeruginosa*, and enhanced gliding of *S*. *aureus*, and 3) isoflurane enhanced biofilm formation by *S*. *aureus*, *E*. *faecalis*, *E*. *coli*, but not *P*. *aeruginosa*, with *S*. *aureus and E*. *faecalis* also being enhanced by sevoflurane. Our results suggest that exposure to volatile anesthetics isoflurane and sevoflurane significantly change the bacterial behaviors and their effects are different among bacteria species.

Swimming is a form of motility driven by flagella and allows bacteria to move toward a favorable environment [37]. From the host defense standpoint, however, flagella are potent stimulators of Toll-like receptor (TLR) 5 molecule and can also be a molecular marker of an invading pathogen for host immune cells. Thus impaired flagella motility could potentially act as protection for bacteria and would allow bacteria to evade host defenses. Several groups found that non-motile isolates of *P*. *aeruginosa* were resistant to macrophage phagocytosis and their presence was associated with poorer clinical scores in patients with cystic fibrosis than the normally motile isolates, which are often found in patients in the early stages of infection [38, 39]. Furthermore, the loss of flagella motility, not the loss of flagella expression was critical for the development of resistance against phagocytosis by immune cells via the activation of PI3K/Akt pathway [40, 41]. In our study, we found that isoflurane and sevoflurane significantly attenuated the swimming motility of *E*. *coli* and *P*. *aeruginosa*. Whether or not this attenuation by volatile anesthetics indicates impairment of phagocytosis by host immune cells and enhances bacterial virulence needs further investigation. In addition, we studied the role of anesthetics on *S*. *aureus* gliding. Our results showed that volatile anesthetic isoflurane increased the motility of *S*. *aureus*. The relationship between motility and virulence in *S*. *aureus* has not been studied and at this point, the clinical implication of our finding remains to be seen.

The formation of biofilm is observed in approximately 80% of bacterial infections and has been recognized to be a contributor in a number of diseases [42]. Biofilm is extremely resistant to clearance by immune cells and to antibiotics. Biofilm-protected cells are 10–1,000 times less susceptible to antibiotics than planktonic cells. Volatile anesthetics significantly enhanced biofilm formation by *S*. *aureus* and *E*. *faecalis* in our study. *S*. *aureus* is one of the most common bacterial pathogens [43]. Bacteremia and skin abscesses are usually caused by planktonic *S*. *aureus*, while osteomyelitis and endocarditis are caused by biofilm-forming strains. *E*. *faecalis*, on the other hand, is responsible for 80–90% of enterococcal infections. *E*. *faecalis* biofilm is important in periodontal infection as well as binding to various medical devices including urethral stents, intravascular catheters, and biliary stents [16]. Given that eradication of *S*. *aureus* and *E*. *faecalis* in biofilm are clinically important challenges to be solved, our data showing that volatile anesthetics facilitated biofilm formation needs to be evaluated further, including *in vivo*.

We also evaluated the growth under anesthetics and we did not find any difference among different anesthetics, which is line with the majority of literature. The study by Molliex et al. showed that the growth of *Pseudomonas aeruginosa* (*P*. *aeruginosa*) was attenuated under isoflurane and halothane exposure [14]. In contradiction, however, we did not observe any differences in *P*. *aeruginosa* as they did. Despite our study sharing similar methods, it was not clear if this difference in results is due to our strain differing from their strain, or culture conditions.

Because ion channels are considered to be prime volatile anesthetic targets and ion transporters are evolutionarily closely related to ion channels in humans [30], we hypothesized that bacterial behaviors were modulated via ion transporters. Taking advantage of multiple single gene deletion mutants that have been developed in *E*. *coli* K12 strains [22], we tested the hypothesis. Isoflurane reduced swimming, enhanced biofilm formation, and did not affect growth in *E*. *coli* parent strain. We identified that several ion transporter mutants showed reduction in swimming, increase in biofilm formation and no change in growth. Our result suggested that *clcB*, *nhaA* and *nhaB* would be isoflurane targets, although there may exist an additional target. We did not show the direct interaction of isoflurane with these molecules. Because specific agonists are not available, a docking simulation using NhaA suggested that isoflurane could bind within this ion transporter. Nearby residues from isoflurane are critical for pH signal transduction as well as serve as the iron binding site, further supporting the idea that isoflurane would affect ion transporter function by interacting its critical sites. The involvement of ion in biofilm formation has also been shown. Prindle et al showed that potassium channel regulates metabolic states for biofilm formation in *Bacillus subtilis* [44]. Therefore, it is possible that volatile anesthetics affected ion movement to modulate bacterial behaviors.

Our findings suggest the potential implication of anesthetic selection for patients with active bacterial infection. Choices of anesthetic drugs could potentially affect the course of infection. So far, the clinical impact of the type of anesthetics on infection has not been well reported. Von Dossow et al. reported their study of alcoholic patients who underwent surgical procedure either with propofol or isoflurane anesthesia and they found that postoperative infection rate was higher in isoflurane group [45]. Certainly the investigation of the *in vivo* impact of anesthetics on bacterial motility and biofilm formation is critical. In addition, identifying the underlying mechanism may allow practitioners to choose anesthetic drugs in a rationalized manner and also provide an opportunity to redesign better anesthetics.

In summary, we have demonstrated that volatile anesthetics modulated bacterial motility and biofilm formation *in vitro* and suggest the potential importance of anesthetic selection in patients with active infection. Future experiments will need to address *in vivo* significance.

## Supporting Information

S1 Fig Motility ring pictures of *E*. *coli* and *P*. *aeruginosa* with withour anesthetics. Swimming ring pictures for *E*. *coli* and *P*. *aeruginosa* were shown. Radius of each plate = 4.25 cm.
(TIFF)

S2 Fig Flagellin expression of ion transporter deletion mutants in *E*. *coli*. Expression of flagellin in ion transporter deletion mutants was probed using anti-flagellin antibody. *fliC* deletion mutant was used as a negative control. GAPDH was used as a loading control.
(TIFF)

S3 Fig The reduction of *E*. *coli* swimming under isoflurane. The percentage of swimming reduction in *E*. *coli* ion transporter deletion mutants by isoflurane exposure was calculated. Statistical analysis was performed using one-way analysis of variance with Bonferroni *post hoc* analysis. * denotes *p* < 0.05 versus parent strain.
(TIFF)

S4 Fig The effect of isoflurane on the double and triple ion transporter deletion mutants. (A) The area of swimming holos from *E*. *coli* K12 parent strain, the double and triple ion transporter deletion mutants. Data are shown as mean +/- S.D. of 4–8 replicates. Statistical analysis was performed using one-way analysis of variance with Bonferroni *post hoc* analysis. ** denotes *p* < 0.01 versus parent strain. (B) The percentage reduction in *E*. *coli* triple deletion mutant by 3% isoflurane was shown. Data are shown as mean +/- S.D. of 8 replicates. Statistical analysis was performed using unpaired student t test. ** denotes *p* < 0.01 versus parent strain.
(TIFF)

S5 Fig The growth of *E*. *coli* K12 parent strain and its ion transporter deletion mutants. The growth of the *E*. *coli* K12 parent strain and its ion transporter deletion mutants is shown. Data are shown as mean +/- S.D. of 4 replicates. Statistical analysis was performed using one-way analysis of variance with Bonferroni *post hoc* analysis. No statistical significance was observed. n.s. = not significant.
(TIFF)
