# Supplementary figures and images for: The Differential Effects of Anesthetics on Bacterial Behaviors
Source: PLoS One. 2017 Jan 18;12(1):e0170089. doi: 10.1371/journal.pone.0170089 (PMC5242519; doi:10.1371/journal.pone.0170089)

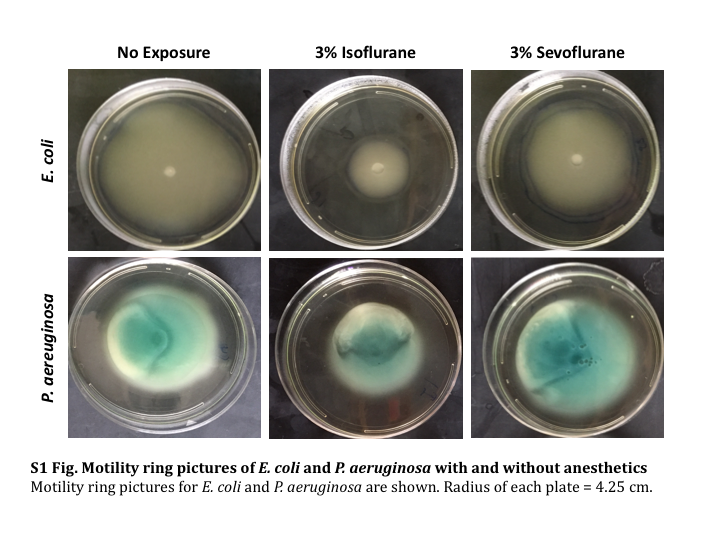

Supplement: S1 Fig — Swimming ring pictures for E. coli and P. aeruginosa were shown. Radius of each plate = 4.25 cm. (TIFF) [file pone.0170089.s001.tiff]

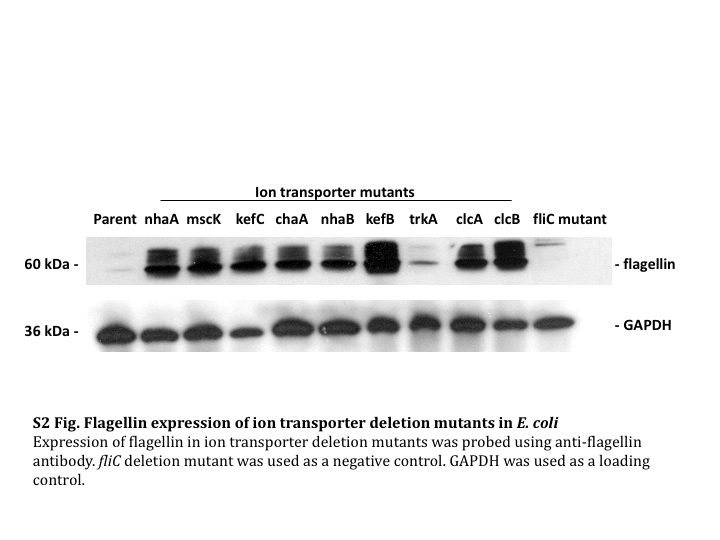

Supplement: S2 Fig — Expression of flagellin in ion transporter deletion mutants was probed using anti-flagellin antibody. fliC deletion mutant was used as a negative control. GAPDH was used as a loading control. (TIFF) [file pone.0170089.s002.tiff]

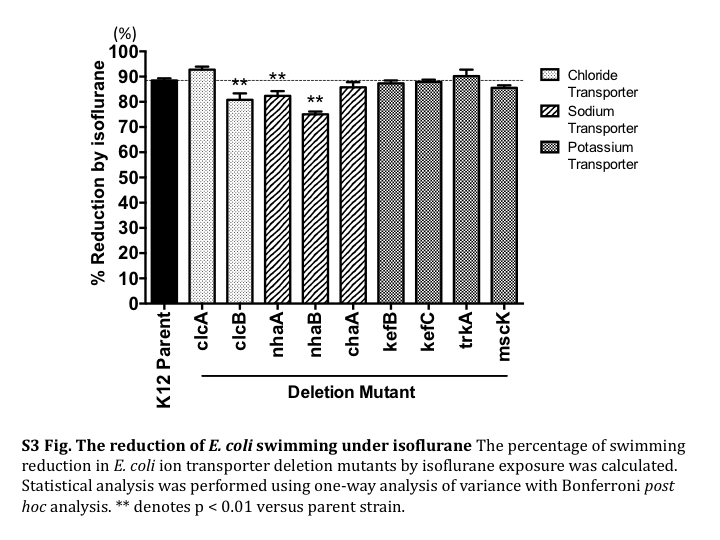

Supplement: S3 Fig — The percentage of swimming reduction in E. coli ion transporter deletion mutants by isoflurane exposure was calculated. Statistical analysis was performed using one-way analysis of variance with Bonferroni post hoc analysis. * denotes p < 0.05 versus parent strain. (TIFF) [file pone.0170089.s003.tiff]

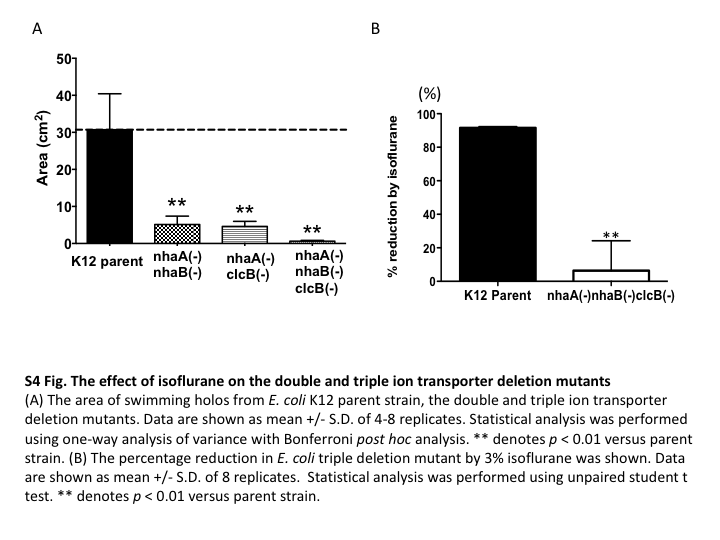

Supplement: S4 Fig — (A) The area of swimming holos from E. coli K12 parent strain, the double and triple ion transporter deletion mutants. Data are shown as mean +/- S.D. of 4–8 replicates. Statistical analysis was performed using one-way analysis of variance with Bonferroni post hoc analysis. ** denotes p < 0.01 versus parent strain. (B) The percentage reduction in E. coli triple deletion mutant by 3% isoflurane was shown. Data are shown as mean +/- S.D. of 8 replicates. Statistical analysis was performed using unpaired student t test. ** denotes p < 0.01 versus parent strain. (TIFF) [file pone.0170089.s004.tiff]

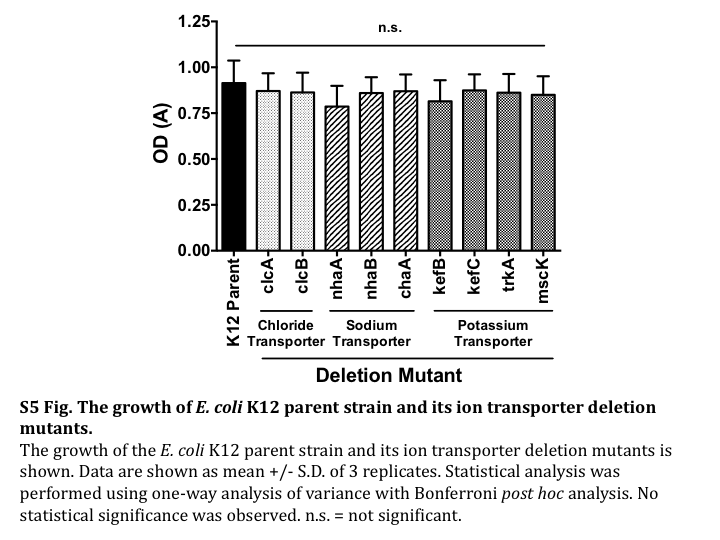

Supplement: S5 Fig — The growth of the E. coli K12 parent strain and its ion transporter deletion mutants is shown. Data are shown as mean +/- S.D. of 4 replicates. Statistical analysis was performed using one-way analysis of variance with Bonferroni post hoc analysis. No statistical significance was observed. n.s. = not significant. (TIFF) [file pone.0170089.s005.tiff]
